# Spexin alleviates insulin resistance and inhibits hepatic gluconeogenesis via the FoxO1/PGC-1α pathway in high-fat-diet-induced rats and insulin resistant cells

**DOI:** 10.7150/ijbs.31781

**Published:** 2019-11-01

**Authors:** Liping Gu, Xiaoying Ding, Yufan Wang, Mingyu Gu, Jielei Zhang, Shuai Yan, Na Li, Zhiyi Song, Jiajing Yin, Leilei Lu, Yongde Peng

**Affiliations:** 1 Department of Endocrinology and Metabolism, Shanghai General Hospital, Shanghai Jiao Tong University School of Medicine, Shanghai, China.; 2 Shanghai Intertek Medical diagnostic Testing Center Co; Ltd, Shanghai 200436, China.; 3 School of Pharmaceutical Engineering& Life Science, Changzhou University, Changzhou, 213164 China.

**Keywords:** spexin, insulin resistance, hepatic gluconeogenesis, FoxO1

## Abstract

**Objective:** Recent studies demonstrate circulating serum spexin levels are reduced in obesity or type 2 diabetes mellitus (T2DM) patients and may play a role in glucose metabolism. The mechanism underlying is not known. In this study, we explore whether spexin has a role in insulin resistance and hepatic glucose metabolism.

**Methods:** The correlation between serum spexin levels and the homeostasis model assessment of insulin resistance (HOMA-IR) was studied in newly diagnosed T2DM patients. After intraperitoneal injection of exogenous spexin for 8 weeks, the effect of spexin on exogenous glucose infusion rates (GIR), and hepatic glucose production (HGP) were assessed by extended hyperinsulinemic-euglycemic clamp in high-fat-diet (HFD)-induced rats. Glucose concentration with CRISPR/Cas9-mediated disruption of spexin expression in HepG2 cells culture was observed. Expression of transcription factors (Forkhead box O1, FoxO1 and peroxisome proliferator-activated receptor gamma coactivator 1-alpha, PGC-1α) and key enzymes (G-6-Pase and PEPCK) of gluconeogenesis pathway were observed in vitro and in vivo.

**Results:** The serum spexin level was significantly low in newly diagnosed T2DM patients as compared with healthy patients and significantly negatively correlated with the HOMA-IR values. Exogenous spexin treatment resulted in weight loss and decrease of HOMA-IR value in high-fat-diet (HFD)-induced rats. The exogenous glucose infusion rates (GIR) were higher in the HFD + spexin group than that in the HFD group (358 ± 32 vs. 285 ± 24 μmol/kg/min, *P* < 0.05). Steady-state hepatic glucose production (HGP) was also suppressed by ~50% in the HFD + spexin group as compared with that in the HFD group. Furthermore, spexin inhibited gluconeogenesis in dose-dependent and time-dependent manner in the insulin-resistant cell model. CRISPR/Cas9-mediated knockdown of spexin in HepG2 cells activated gluconeogenesis. Moreover, spexin was shown regulating gluconeogenesis by inhibiting FoxO1/PGC-1α pathway, and key gluconeogenic enzymes, (PEPCK and G-6-Pase) in both HFD-induced rats and insulin-resistant cells.

**Conclusions:** Spexin plays an important role in insulin resistance in HFD-induced rats and insulin-resistant cells. Regulation of the effects of spexin on insulin resistance may hold therapeutic value for metabolic diseases.

## Introduction

The incidence of metabolic diseases, such as obesity, diabetes, and metabolic syndrome, has increased in recent years. Insulin resistance is a feature of metabolic syndrome. It is strongly associated with dyslipidaemia, obesity, non-alcoholic fatty liver disease (NAFLD) and hyperglycaemia, and type 2 diabetes mellitus (T2DM)^1^. Hepatic insulin resistance is a key factor related to many obesity-associated pathophysiological conditions such as diabetes and NAFLD. Unlike the insulin resistance of muscle and adipose which occur years before the onset of hyperglycaemia and remain relatively stable throughout the course of the disease^2^, the increase of hepatic glucose production (HGP) occurs 'late' in the history of diabetes but worsen progressively and is often refractory to treatment^3^. In T2DM, HGP is higher in the post-absorptive state and fails to be properly suppressed by insulin, which results in primarily from excessive gluconeogenesis rather than glycogenolysis^4^. Several factors contribute to the elevated gluconeogenesis that observed in diabetes, including (i) increased supply of glucogenic precursors to the liver (glycerol, amino acids, and free fatty acids (FFA)); (ii) vagal control originating from the rodent hypothalamus; and (iii) decreased insulin receptor signalling activity in hepatocytes 5.

Gluconeogenesis is primarily modulated by phosphoenolpyruvate carboxykinase (PEPCK) and glucose-6-phosphatase (G-6-Pase)^6^. PEPCK and G-6-Pase are up-regulated in response to hormones during fasting, and are robustly down-regulated by insulin and glucose 7, 8. They are crucial enzymes that convert pyruvate to glucose, and their gene expression levels are regulated by several transcription factors, such as Forkhead box O1 (FoxO1), cAMP response element binding protein (CREB), hepatocyte nuclear factor 4 (HNF4), glucocorticoid receptor, and peroxisome proliferator-activated receptor gamma coactivator 1-alpha (PGC-1α) 9-11. In the liver, FoxO1 is activated during fasting and is inactivated by feeding under physiological conditions, which is one of the essential mechanisms by which insulin represses HGP rapidly and efficiently during postprandial periods 12-14. In insulin resistance, phosphorylated FoxO1 will enter the cell nucleus and bind to PGC-1α, which induces the expression of target genes that are responsible for gluconeogenesis in the liver, such as G-6-Pase and PEPCK genes 15.

Spexin is a recently identified neuropeptide composed of 14 amino acids 16, 17. Previous studies have revealed that there are widespread expression of spexin mRNA and protein in the endocrine system of both rats and humans 18, 19. Its high expression in endocrine cells and the ability to be secreted suggest that spexin may function as an endocrine factor. Circulating spexin levels change in parallel and negatively correlate with the leptin levels in humans (*r* = -0.797). In diet-induced obese rats, intraperitoneal injection of spexin (35μg/kg/day) for 19 days can reduce the caloric intake by 32%, along with corresponding weight loss 20. Recently, we found that spexin may be involved in regulating glucose metabolism 19. One of our studies showed that the serum spexin level was significantly reduced in T2DM patients as compared with that in control group. Furthermore, oral glucose tolerance test (OGTT) in 12 healthy volunteers shows a negative correlation between the serum spexin level and blood glucose level during the time scale of OGTT 19. Therefore, spexin might be involved in the regulation of glucose metabolism.

The aim of the present study was to examine whether treatment with spexin peptide could affect insulin resistance and HGP in vivo and in vitro. Here, for the first time, we provide evidence demonstrating that spexin inhibits hepatic gluconeogenesis to alleviate insulin resistance in high-fat-diet (HFD)-induced rats and insulin-resistant cells via the FoxO1/PGC-1α pathway.

## Methods

### Subjects and OGTT

This study included 45 patients with an average age of 54.0 ± 15.3 years (23 patients were newly diagnosed with T2DM while 22 were with normal glucose levels). None of the patients were receiving drug or insulin therapy before the blood test and no patients had a family history of diabetes. Exclusion criteria for the study were as follows: type 1 diabetes, chronic hepatic disease, renal failure, autoimmune diseases, malignant diseases, and pregnancy. Informed consent was obtained from all participants before enrolment. The study was approved by the Institutional Review Board of Shanghai First People's Hospital affiliated to Shanghai Jiao Tong University School of Medicine (No. 2014KY094) and was performed in accordance with the principles of the Declaration of Helsinki.

Briefly, all patients were administered 75 g of glucose at 8:00 A.M. after an overnight fast for at least 8 hours. The plasma glucose and insulin levels were measured at 0, 1 and 2 h after fasting. All blood samples were drawn from the antecubital vein. For each subject, the body mass index (BMI) was calculated at the time of blood collection as weight in kilograms divided by height in metres squared. Plasma glucose concentrations were analysed with the glucose oxidase method. The insulin levels were measured using a radioimmunoassay (RIA) kit (cat. no. F01PZA; Beijing North Institute of Biological Technology, Beijing, China). Serum glycosylated haemoglobin (HbAlc) levels were measured by anion exchange high-performance liquid chromatography (Arkray Inc., Shanghai, China). Triglycerides (TG), total cholesterol (TC), low-density lipoprotein cholesterol (LDL-C), and high-density lipoprotein cholesterol (HDL-C) were measured using an autoanalyser (Beckman, CA, USA). Serum spexin levels were determined using enzyme-linked immunesorbent assay (ELISA) (cat. no. EK-023-81 CE; Phoenix Pharmaceuticals, Belmont, CA, USA). The homeostasis model assessment of insulin resistance (HOMA-IR) was calculated using the following equations: HOMA-IR = (FBG (mmol/L) × FIN (μIU/mL)) / 22.5^21^.

### Animal protocol

Healthy male Sprague-Dawley rats (6 weeks old, 150-200 g) were obtained from Shanghai Slac Laboratory Animal Co. Ltd. (License No, SCXK (Shanghai) 2013-0016) and housed at 20-22°C on a 12 h light/12 h dark cycle and allowed to feed ad libitum. The animals were randomly assigned to either the control group or HFD intervention group. The control group was fed with regular rat chow (62% carbohydrate, 27% protein, and 11% fat), while the HFD group was provided with the HFD (45% carbohydrate, 20% protein, and 35% fat, Slac Laboratory Animal Co. Ltd., Shanghai, China). The rats in the control group were assigned either to treatment with phosphate buffered saline (PBS) (control group, n = 6) or spexin (control + spexin, n = 6). The HFD rats were also assigned to one of the two treatments: the PBS-treated group (HFD group, n = 6) or the spexin-treated group (HFD + spexin, n = 6). The spexin intervention (a custom synthesis product from SBS Genetech Co., Ltd., Beijing, China) was initiated at 18 weeks of age and continued for 8 weeks. Spexin was administered by subcutaneous injection at a dose of 50μg/kg/day. The rats in control group of were given the same volume of PBS as that in spexin-treated group every day. Animals were fasted overnight before the experiments. Testing was conducted during the light cycle in free-running rats, with all protocols initiated between 08:00 and 09:00. All animal experimental protocols were according to the guide for the care and use of laboratory animals (8^th^ edition) and all experimental protocol was approved by the Institutional Animal Care and Used Committee, Shanghai Jiaotong University School of Medicine, Shanghai, China (No. 1020150511).

At the age of 26 weeks, blood samples were collected from the tail vein before and at 30, 60, and 120 min after the intraperitoneal injection of 2 g/kg glucose (50% dextrose). Spexin was measured on the fasting samples. The glucose area under curve (GAUC) was calculated using the following trapezoidal method: GAUC = 1/2(FPG + GLU30) × 30 + 1/2(GLU30 + GLU60) × 30 + 1/2(GLU60 + GLU120) × 60. The fasting serum insulin levels were measured by radioimmunoassay (Tosoh, Tokyo, Japan). Serum TG, TC and FFA were determined using commercially available kits according to the instructions of the kits (Jian Cheng Biological Engineering Institute, Nanjing, China).

The hyperinsulinemic-euglycemic clamp technique was performed as previously described 22. After an overnight fast of 12 h, the rats were anesthetized with intraperitoneal injection of pentobarbital sodium (40 mg/kg). One cannula was inserted into the right jugular vein to infuse glucose and insulin, and another cannula was inserted into the left carotid artery for blood sampling. D-[^13^C_6_]-Glucose (cat. no. S14374 IsoScience, PA USA) was administered at a one-time intravenous (i.v.) dosage of 10 mg, followed by continuous infusion through the i.v. infusion line driven by a mini-infusion pump (Injectomat Agilia, Brezins, France) for 60 min (basal period). The infusion rate was 0.1 mg/kg/min. During the infusion of D-[^13^C_6_]-Glucose, the glucose clamp was initiated by infusing insulin at a constant rate of 4.8mU/kg/min until the end of the clamp for an additional 120 min (chase period). Thereafter, a 25% glucose solution was simultaneously infused at a variable rate to maintain plasma glucose values at the clamp level (5.0 ± 0.5mmol/L). In all studies, blood samples were obtained to determine the insulin levels at -90, 0, 80, 100, 110, and 120 min. The samples were prepared on ice, centrifuged at 4°C, separated, and stored at -80°C until the time of measurement. Afterward, portions of each liver were fixed in 10% buffered formalin for histopathological examination, and other lobules of liver were quickly frozen in liquid nitrogen for biochemical analyses.

### Gas Chromatography-Mass Spectrometry (GC-MS) analysis and calculations of clamp-related indexes

Enrichment of the glucose concentrations was measured by GC-MS analysis of the trimethylsilyl derivatives. Briefly, 1 μl of each sample was injected (split-less mode) into the gas chromatograph (Agilent 7890-5975C, Palo Alto, CA USA) equipped with a 30 m fused silica capillary column (DB-5MS, Agilent, Palo Alto, CA USA) and interfaced with a mass spectrometer (Agilent, Palo Alto, CA USA) operating in the electronic impact ionization mode. The carrier gas was helium. The operating conditions were as follows: injector at 270°C and oven at 180°C and then increased to 280°C at 5°C/min for 20 min. Ions with mass-to-charge ratios (m/z) of 314 (unlabelled glucose) and 319 (labelled D-[^13^C_6_]-glucose) were selectively monitored. The 314/319 peak area ratio was calculated and the corresponding enrichment was determined.

An estimate of the whole-body glucose utilization rate and hepatic glucose production were calculated as previously described 23. In the steady-state clamp, the GIR represented the insulin sensitivity of the body. The glucose disappearance rate (GRd) was calculated using the steady-state equation from the respective tracer infusion rates and enrichment (expressed as specific glucose activity). In the basal state, HGP is equal to GRd after an overnight fast. The detailed formulas were as follows: GRd = D-[^13^C_6_]-glucose infusion rate/D-[^13^C_6_]-glucose enrichment at the steady state, HGP = D-[^13^C_6_]-glucose infusion rate/D-[^13^C_6_]-glucose enrichment at 0 min.

### Histopathological examination

Formalin-fixed liver samples were embedded in paraffin, cut into 5-μm slices, and then stained with H&E, according to the manufacturer's instructions. Images of the stained tissues were acquired using a light microscope (Leica DM4000B, Solms, Germany). The sections were microscopically examined by two histopathologists. Liver sections from each group were blindly analyzed and assigned one of five scores of severity of hepatocellular lesions in 3 random microscopic fields (×200 magnification) per section (one section per rat): 0, none; 1, minimal, defined as only occasional vacuolar degeneration in any lobule; 2, mild, defined as less than one-third of the lobular structure affected; 3, moderate, defined as between one-third and two-thirds of the lobular structure affected; 4, severe, defined as greater than two-thirds of the lobular structure affected.

### Cell culture and treatment

The HepG2 human hepatic carcinoma cell line was purchased from the Cell Bank of Chinese Academy of Sciences (Shanghai, China). The cells were cultured and maintained at 37°C and 5% CO_2_ in DMEM with 10% foetal bovine serum (FBS), 2 mM glutamine, penicillin, and 1% streptomycin. A primary culture of mouse hepatocytes was prepared as previously described^24^. Cells from the liver of 8 weeks old wild type mice (Shanghai Slac Laboratory Animal Co. Ltd., License No, SCXK (Shanghai) 2013-0016) were seeded at a density of 1 × 10^6^/well onto 60 mm culture plates in plating medium consisting of DMEM/F12 medium supplemented with 15% FBS and penicillin/streptomycin.

The insulin-resistant cell model was induced using modified methodas previously described 25, 26. Briefly, cells were treated with various concentrations of insulin (0.1, 1, 5, 10, 50, and 100 μg/mL) (cat. no. 11061-68-0; Sigma-Aldrich, St. Louis, MO USA) for 36 h. The glucose concentrations in the supernatants were measured with a glucose oxidase kit (cat. no. MAK097-1KT, Sigma-Aldrich, St. Louis, MO USA). The appropriate concentration of insulin was selected by measuring the glucose concentration and was used for all subsequent experiments. Next, the cells were treated with the optimum concentration of insulin for 12, 24, 36, and 48 h to induce IR. The cell culture supernatant was collected to measure the glucose concentration. Cell viability was measured using a cell counting kit-8 (CCK-8, cat. no. CK04-11; Dojindo Laboratories, Kumamoto, Japan) according to the manufacturer's instructions. Then, the effects of spexin (cat. no. 023-81; Phoenix Pharmaceuticals, Belmont, CA USA) were tested. All measurements were conducted in triplicate.

### Immunocytochemistry

Cells were seeded onto 24-well culture plates at a density of 75,000 cells per well and grown in a 5% CO_2_ atmosphere at 37°C for 24 h. Then, the cells were fixed with 4% paraformaldehyde in PBS for 10 min. Next, the cells were permeabilized in situ with 0.2% Triton X-100 in PBS for 10 min, and then non-specific binding was prevented by incubation with 1% bovine serum albumin in PBS (blocking solution) for 1 h. Subsequently, the cells were incubated with appropriate dilutions of anti-spexin rabbit polyclonal primary antibody (cat. no. ab121385; Abcam, Cambridge, UK) in a humidified chamber at 4°C overnight. After incubation, the cells were washed three times (15 min each) with PBS. Then, they were incubated with secondary antibodies against IgG (cat. no. 111-515-003; Jackson ImmunoResearch, West Grove, PA, USA) at a 1:100 dilution and with 10 μg/mL DAPI (cat. no. C1006; Beyotime Biotechnology, Shanghai, China) at room temperature for 2 h. The cells were analysed with a Leica SP8 confocal fluorescent microscope (Leica, Wetzlar, Germany).

### Spexin gene disruption in HepG2 cells and siRNA-mediated knockdown

HepG2 cells were seeded into 6-well tissue culture plates (2.5×10^3^/well) and grown to 80% confluency in antibiotic-free normal growth media containing FBS. Knockdown was performed using CRISPR/Cas9 spexin gene disruption materials. Briefly, a spexin-specific or control pool of CRISPR/Cas9 sgRNA plasmids (constructed by Shanghai Taiting Bioscience Co., Ltd, Shanghai, China) was cotransfected with the spCas9-expressing vector (cat. no. wx141; Taiting Bioscience Co. Ltd., Shanghai, China); the former plasmids allowed for puromycin selection of cells that were transfected with the knockout plasmid. Transfections used a total of 3 μg of plasmid (sgRNA:spCas9 = 1:1) and the Lipofectamine2000 transfection reagent (cat. no. 11668-019; Invitrogen, Carlsbad, CA, USA), according to the manufacturer's recommendations. After puromycin selection, the cells were cultured in normal growth media and allowed to reach 90% confluency prior to subsequent assays, including evaluations of gene silencing, which was confirmed by ELISA and qRT-PCR.

HepG2-IR Cells were transfected with siRNA following manufacturer's instructions by electroporation. The siRNA for FOXO1 was purchased from Dharmacon (cat. no.D003006060020, Dharmacon Inc, shanghai, China). The cells were harvested 48 h after transfection.

### RNA preparation and quantitative real-time PCR analysis

Total RNA was extracted from liver tissues or cells with TRIzol reagent (cat. no. 15596018; Life Technologies, Invitrogen, Carlsbad, CA, USA) according to the manufacturer's instructions. Subsequently, 1 μg of total RNA was reverse-transcribed into cDNA using a Reverse Transcription system (cat. no. 18080051; Invitrogen, Carlsbad, CA, USA). Quantitative real-time PCR was then performed in duplicate using the SYBR premix Ex Taq kit (cat. no. 4367659; Applied Biosystems, Foster City, CA, USA) and Applied Biosystems ViiA^TM^ 7 machine (Life Technologies, Burlington, ON, USA). The primer sequences were listed in Supplementary Table S1. The obtained results were normalized to GAPDH which was used as a reference gene. The data were analysed using the 2 ^(-delta delta Ct)^ methods.

### Western blot analysis

Liver tissues and cells were washed with PBS and lysed in lysis buffer (0.5% Triton X-100, 10 mM HEPES, pH 7.9, 50 mM NaCl, 100 mM EDTA, and 0.5 M sucrose) containing 0.1% protease inhibitor cocktail (cat. no. 4693132001; Sigma-Aldrich, St. Louis, MO, USA). The lysates were then incubated on ice for 30 min and centrifuged at 8,000 ×*g* for 10 min. Equal amounts of protein were subjected to SDS-PAGE (10-15%), transferred to polyvinylidene difluoride membranes, and immunoblotted with the following primary antibodies: anti-FoxO1 (256) antibody (cat. no. SAB4300094; Sigma-Aldrich, St. Louis, MO, USA), anti-FoxO1 antibody (cat. no. SBP3500507; Sigma-Aldrich, St. Louis, MO, USA), anti-PGC-1α antibody (cat. no. 2178S; Cell Signaling Technology, Danvers, MA, USA), anti-PEPCK antibody (cat. no. 8565S; Cell Signaling Technology, Danvers, MA, USA), anti-G-6-Pase antibody (cat. no. SAB1303137; Sigma-Aldrich, St. Louis, MO, USA), anti-CREB antibody (cat. no. 9197; Cell Signaling Technology, Danvers, MA, USA), anti-phospho-CREB (Ser133) antibody (cat. no. 9198; Cell Signaling Technology, Danvers, MA, USA), anti-Akt antibody (cat. no. 4685; Cell Signaling Technology, Danvers, MA, USA), anti-phospho-Akt (Ser473) antibody (cat. no. 4060; Cell Signaling Technology, Danvers, MA, USA), anti-HNF-4α (Hepatocyte nuclear factor 4α) antibody (cat. no. 3113; Cell Signaling Technology, Danvers, MA, USA), and anti-PPARα (Peroxisome proliferator-activated receptor α) antibody (cat. no. ab126285; Abcam, Cambridge, Massachusetts, UK). The membranes were washed with PBS-Tween-20 and incubated with a peroxidase-conjugated secondary antibody. The protein bands were detected using an ECL Plus kit (Amersham Biosciences Corp., Piscataway, NJ, USA).

### Statistics

Statistical analyses were performed using the SPSS statistical software for Windows (Version 20.0). Numerical data are presented as the means ± SD. Data with a skewed distribution were logarithmically transformed before the analysis. Comparisons between groups were performed using independent-samples t tests, paired-samples t tests or one-way analysis of variance (ANOVA) or two-way ANOVA. A chi-square test was used to analyse categorical data, and descriptive statistics are presented as frequencies (%). Correlation analyses were adjusted by age and body weight and were performed using Pearson's test or Partial's test. *P* values of less than 0.05 were considered statistically significant.

## Results

### Serum spexin levels in T2DM and their correlation with HOMA-IR

The T2DM group and control group did not differ in age (55.3 ± 13.2 vs. 52.7 ± 17.4 years, *P* > 0.05) or BMI (23.5 ± 2.8 vs. 22.3 ± 2.6, *P* > 0.05). However, there was a significant difference in the fasting blood glucose level (FBG, *P* < 0.01), postprandial blood glucose level (PBG1h, PBG2h, *P* < 0.01), HbA1c level (*P* < 0.01), postprandial insulin level (PINS2h, *P* < 0.01), TG (*P* < 0.01), LDL-C (*P* < 0.01) and homeostasis model assessment of insulin resistance (HOMA-IR) (*P* < 0.01)(Table 1). The serum spexin levels in the T2DM group were decreased by 56% as compared with those of the control group (1.69 ± 1.07 ng/mL vs. 3.82 ± 1.54 ng/mL, *P* < 0.01). Furthermore, there was a significant correlation between the serum spexin level and FBG (*r* =-0.583, *P* < 0.01), PBG1h (*r* =-0.457, *P* < 0.01), PBG2h (*r* =-0.502, *P* < 0.01), HbA1c (*r* =-0.431, *P* < 0.01) and HOMA-IR (*r* =-0.498, *P* < 0.01) after adjusting for BMI. No significant correlations between serum spexin levels and TG (*r* = - 0.180), TC (*r* = - 0.191), LDL-C (*r* = - 0.270) and HDL (*r* = - 0.033) were observed. These results showed strong negative correlations between the serum spexin level, glucose level and HOMA-IR in the OGTT, suggesting that spexin is related to insulin resistance.

### Effect of exogenous spexin on body weight, blood parameters, and HOMA-IR in HFD-induced SD rats

**Figure 1:**
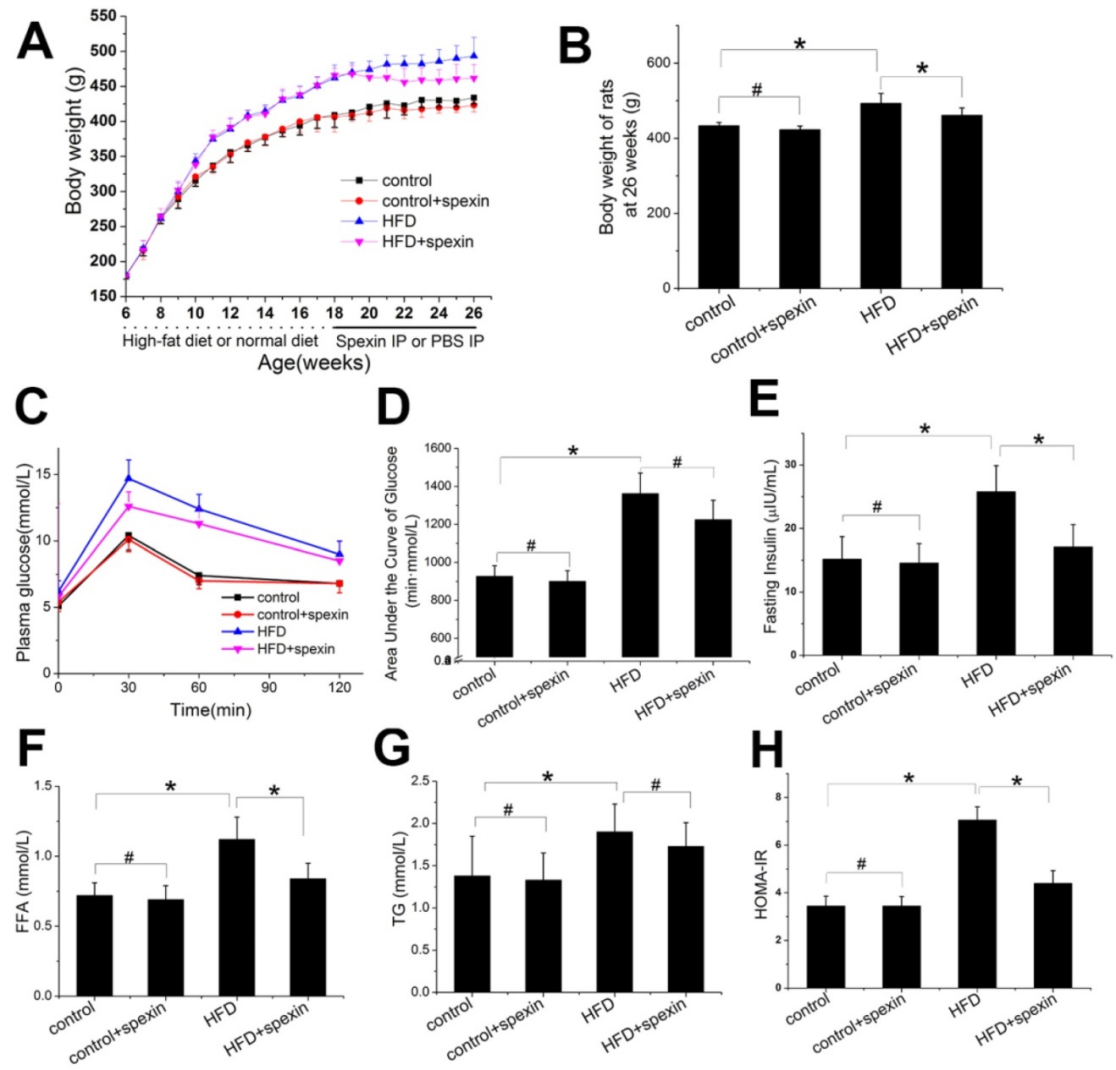
** Effect of exogenous spexin on body weight, blood parameters, and HOMA-IR in HFD-induced SD rats.** (A) Effect of exogenous spexin on body weight in HFD and normal diet rats. (B) Changes of the body weights of SD rats in HFD and normal diet rats at the age of 26 weeks. (C) Changes of the blood glucose levels in SD rats during the IPGTT. (D) Analysis of the AUCG in the IPGTT of SD rats. (E, F, G) Effect of Spexin on fasting insulin level, FFA, and TG in HFD-induced SD rats. (H) Effect of spexin on HOMA-IR in HFD-induced SD rats. Statistical differences between individual groups were evaluated using Student's *t* test (*, *P* < 0.05; #, *P* > 0.05).

HFD feeding for twelve weeks significantly increased the body weight and fasting plasma glucose concentrations as compared with animals fed with control diet (Supplementary Fig. S1A). After 12 weeks of HFD feeding, the fasting plasma insulin concentrations were significantly increased and were approximately 1.7 times higher than before. As shown in Fig. 1A, during the period of spexin treatment, the body weight curve of the HFD + spexin group and the HFD group was gradually separated, while the difference was not significant between the spexin+control groups and the control group. After 8 weeks treatment of spexin, the body weight of the rats at 26 weeks of age in the HFD + spexin group was significantly lower than those in the HFD group (*P* < 0.05) (Fig. 1B). Exogenous spexin intervention had no significant effect on blood glucose and the area under the curve of glucose (*P* > 0.05, Fig. 1C, D). Spexin reduced the insulin concentrations in the HFD-fed rats, while no obvious changes were observed in the normal diet-fed group (Fig. 1E). However, Spexin treatment reduced the serum FFA levels (both P < 0.01) but not the serum TG level, when compared with the levels observed in the HFD group (Fig. 1F, G). Exogenous spexin also reduced the HOMA-IR value in the HFD-fed rats but not normal diet-fed rats (Fig. 1H).

### Exogenous spexin decreased liver glucose production based on the hyperinsulinemic-euglycemic clamp test

**Figure 2:**
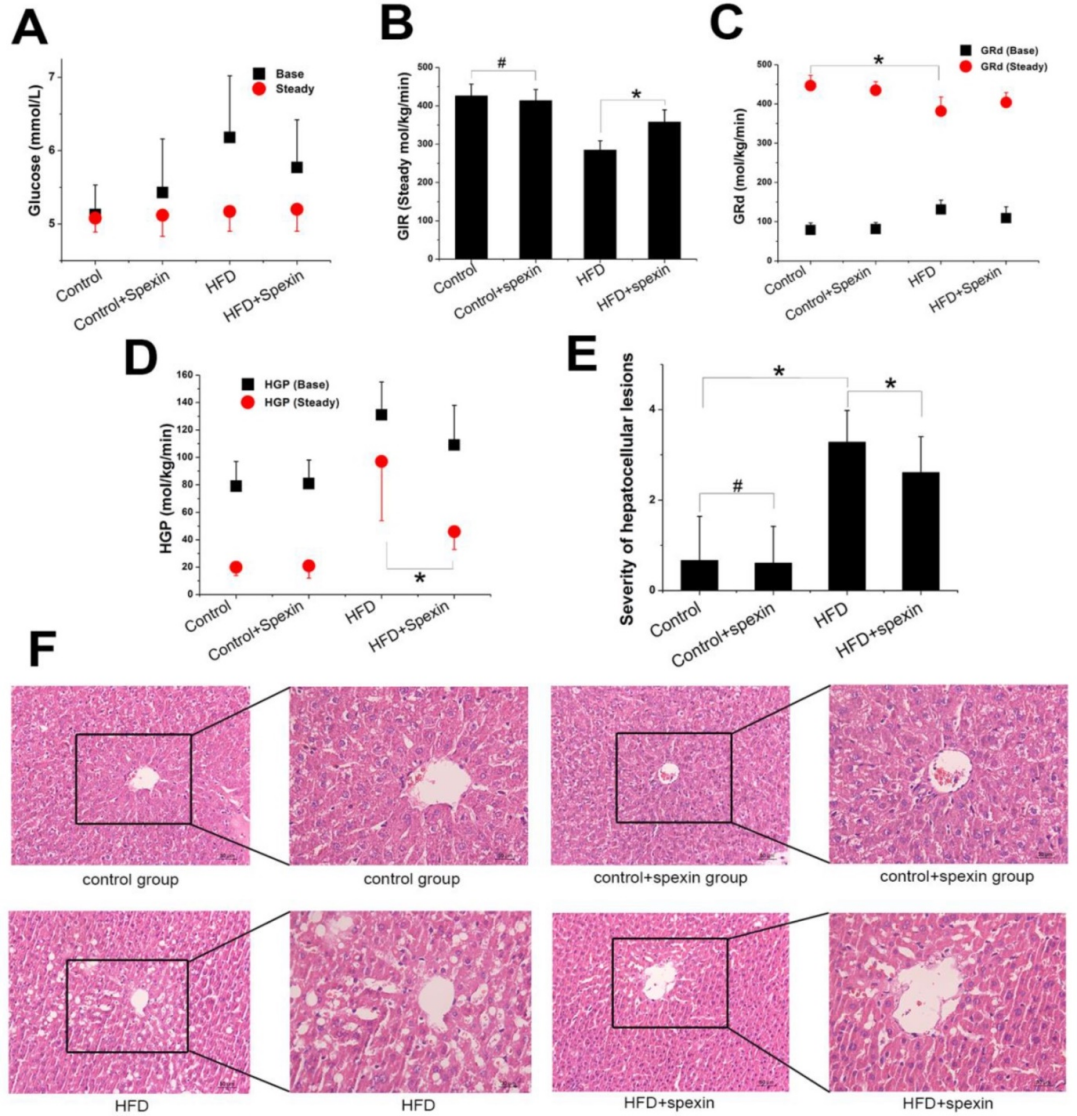
** Glucose metabolism parameters of HFD-induced IR rats in the hyperinsulinemic-euglycemic clamp test and liver morphology shown by H&E staining.** (A) The serum glucose levels in the HFD + spexin group was much lower than that in the HFD group as determined by the clamp experiment. (B) The exogenous GIR required for maintaining glucose levels at the clamp point were higher in the HFD + spexin group than that in the HFD group. (C) After a 90-min exogenous insulin infusion, the GRd of the four groups was significantly increased as compared with that of the basal state, particularly in the control group. (D) The steady-state HGP was suppressed by ~50% in the HFD + spexin group as compared with that of the HFD group. (E) The scores of severities of hepatocellular lesions in the HFD + spexin group were remarkably lower than that in the HFD group. (F) Exogenous spexin improved hepatic steatosis in HFD-induced IR rats. (* *P* < 0.05, # *P* > 0.05. Scale bars are 50 μm and 30 μm, respectively).

The exogenous glucose infusion rates (GIR) required for maintaining glucose levels at the clamp point (5-5.5 mmol/L) were higher in the HFD + spexin group than those in the HFD group (358 ± 32 vs. 285 ± 24 μmol/kg/min, *P* < 0.05), although these rates were still significantly reduced as compared with those of normal diet fed group (*P* < 0.05). The GIR did not differ between the control group and the control + spexin group (426 ± 30 vs. 414 ± 28 µmol/kg/min) (Fig. 2B). The baseline glucose disappearance rates (GRd) of the HFD + spexin group were lower than those in the HFD group. But they were still significantly increased as compared with those of the control + spexin group orthe control group (*P* < 0.05). After a 90-min exogenous insulin infusion, the GRd of the four groups was significantly increased as compared with the baseline GRd, particularly in the control group. In the steady state, there were significant differences in the HGP among the four groups. The steady-state HGP was suppressed by ~50% in the HFD + spexin group as compared with that of the HFD group (46 ± 13 vs. 97 ± 43 µmol/kg/min, *P* < 0.01) (Fig. 2C). The rate of HGP inhibition in the control group was more than 80%, whereas the HGP of the HFD group was not completely inhibited (inhibition rate of only 26%). The basal HGP was significantly increased in the HFD group as compared with that of the control group (131 ± 24 vs. 79 ± 18 µmol/kg/min, *P* < 0.05) (Fig. 2D). Insulin-mediated suppression of HGP during physiological hyperinsulinemia was decreased from the basal levels in all groups.

### Exogenous spexin improved hepatic steatosis

Rat liver cell morphology was analysed by haematoxylin and eosin (H&E) staining (Fig. 2E, F). In the control and control + spexin groups, the hepatic cords were radially arranged around a central vein with few lipid droplets. In the HFD group, the lobular structure was destroyed and widely distributed with more lipid droplets than the control group. In the HFD + spexin group, the lipid droplets decreased and were smaller in size in comparison to the HFD group. The scores of severities of hepatocellular lesions in the HFD + spexin group were remarkably lower than that in the HFD group (*P* < 0.05). Furthermore, no adverse effects, such as hepatocyte inflammation, necrosis, or haemorrhage were observed in the liver tissues of rats treated with spexin. These findings suggested that exogenous spexin improved the hepatic steatosis of insulin resistant rats induced with HFD.

### Spexin inhibited HGP in HFD-induced IR rats via the FoxO1/PGC-1α signalling pathway

**Figure 3:**
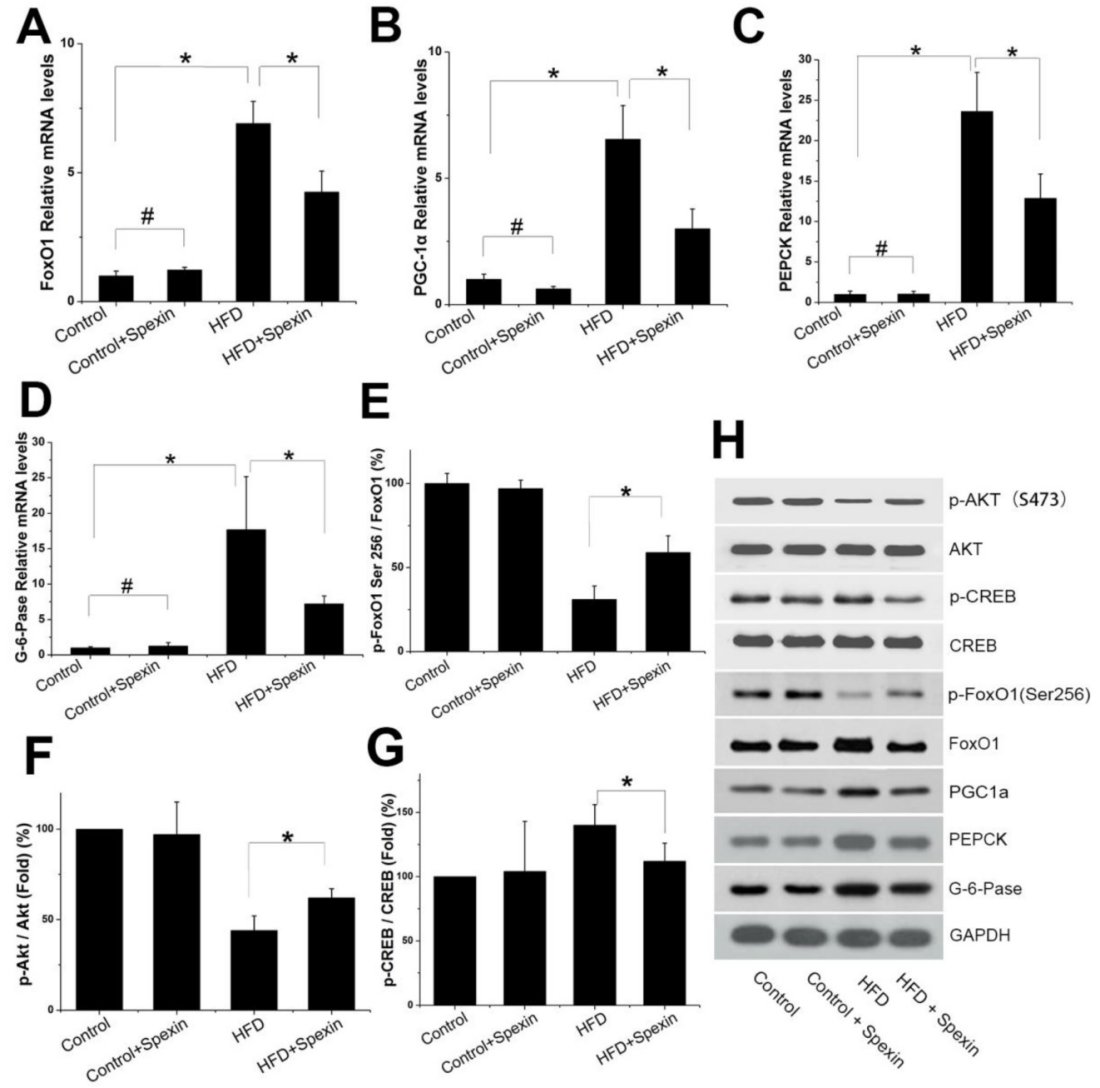
** Expression of Akt, CREB, FoxO1, PGC-1, PEPCK, and G-6-Pase in the liver tissues of rats.** (A, B, C, D) The mRNA expression of FoxO1, PGC-1α, PEPCK, G-6-Paseα in the HFD + spexin group was significantly decreased as compared with that in the HFD group. (E) The spexin treatment increased the p-FoxO1 Ser256/FoxO1 ratio. (F) The spexin treatment increased the p-Akt /Akt ratio. (G) The intervention of spexin decreased the p-CREB /CREB ratio. (H) The protein expression of p-Akt, Akt, p-CREB, CREB, FoxO1, PGC-1, PEPCK, and G-6-Pase. * *P* < 0.05, # *P* > 0.05.

We further measured the mRNA and protein expression of FoxO1, PGC-1α, PEPCK and G-6-Pase in the livers of rats by qRT-PCR and Western blotting, respectively. The FoxO1/PGC-1α signalling pathway was found to be active in the HFD group. Exogenous spexin significantly inhibited the expression of FoxO1, PGC-1α, PEPCK, and G-6-Pase (Fig. 3 A-D, *P* < 0.05), although no significant difference in expression was observed between the control group and control + spexin group (*P* > 0.05). The p-FoxO1 Ser256/FoxO1 ratio was increased in the HFD + spexin group as compared with that in the HFD group (Fig. 3E, F). The increase in the p-FoxO1 Ser256/FoxO1 ratio suggested an increase of FoxO1 phosphorylation and its translocation from the nucleus to the cytoplasm. Therefore, the expression levels of PEPCK and G-6-Pase which were key enzymes for hepatic gluconeogenesis were reduced following spexin treatment. Our results also showed that the ratio of p-Akt/Akt in the HFD group was significantly lower than that in the control group and spexin treatment significantly increased the p-Akt/Akt ratio expression (*P*<0.05) (Fig. 3). The expression of the phosphorylated form of CREB was significantly suppressed in the HFD+spexin group than that in HFD group (*P*<0.05) (Fig. 3).

### Spexin inhibited gluconeogenesis in insulin resistant (IR) cells in a dose and time dependent manner

**Figure 4:**
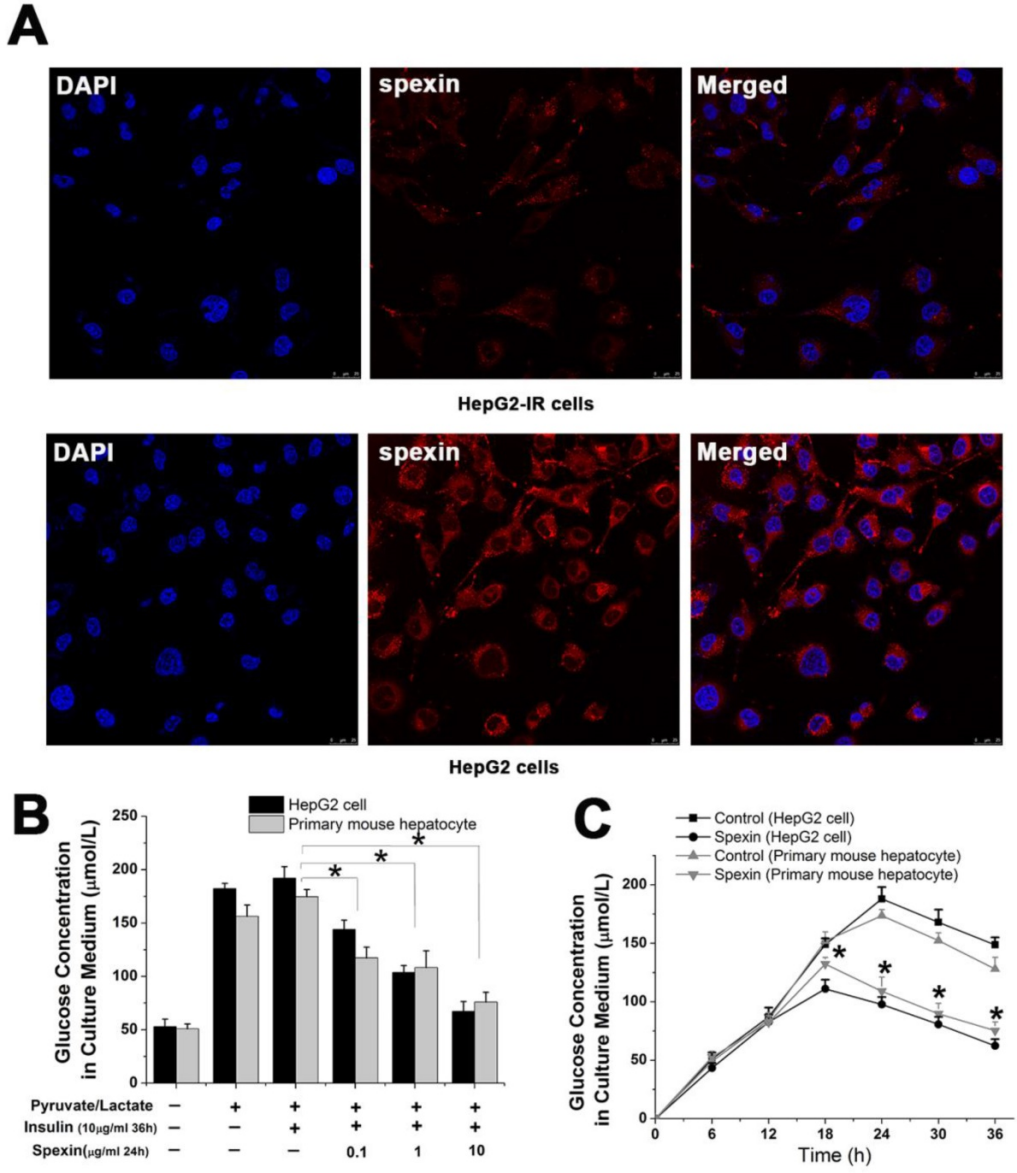
** Spexin inhibited gluconeogenesis in a dose- and time dependent manner in an IR cell model.** (A) Spexin was mainly expressed in the cytoplasm of the HepG2 cells. The spexin expression was significantly reduced in HepG2-IR cells as compared with that in the control group. (B) HepG2-IR cells and primary mouse hepatocytes were cultured with different concentrations of spexin. The higher the concentration of spexin was correlated to a lower glucose concentration in the supernatant. (C) Endogenous glucose production was significantly decreased in HepG2-IR and primary mouse hepatocytes -IR cells that were cultured with 1 μg/mL spexin for 18 h, 24 h, 30 h, and 36 h compared with that of the control group.* *P* < 0.05.

We successfully established an IR cell model by incubating HepG2 cells with 10 μg/mL insulin for 36 h (Supplementary Fig S2 A). Notably, spexin expression was reduced in the cytoplasm of HepG2-IR cells as compared with that in normal HepG2 cells (*P* < 0.05) (Fig. 4A). The glucose concentration in the culture medium reflected the level of gluconeogenesis in HepG2 cells. Both primary mouse hepatocytes and HepG2 cells were incubated with different concentrations of spexin. It was shown that higher spexin concentrations and longer durations of spexin action further decreased the glucose contents in the supernatant (Fig. 4B, C).

### Spexin incubation inhibited gluconeogenesis in insulin-resistant cells via the FoxO1/PGC-1α signalling pathway

**Figure 5:**
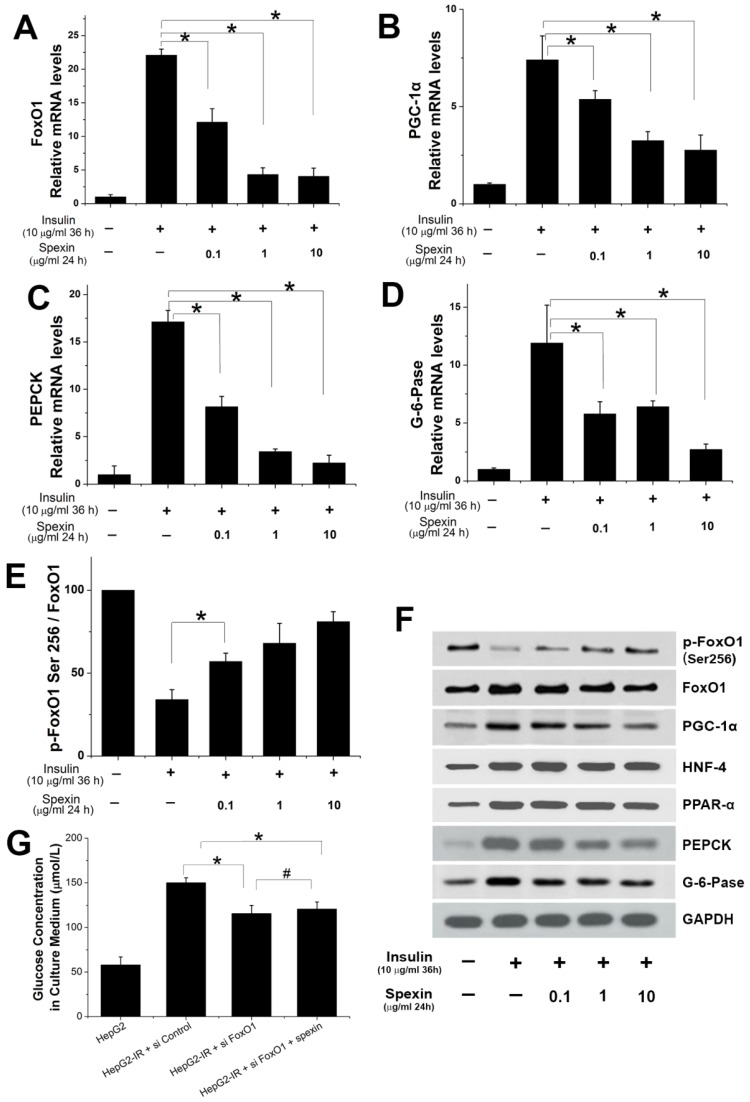
** Expression of important transcription factors and key enzymes in gluconeogenesis.** (A, B, C, D) Spexin dose-dependently inhibited the expression of FoxO1, PGC-1α, PEPCK, G-6-Pase mRNA. (E) The spexin treatment increased the p-FoxO1 Ser256/FoxO1 ratio. (F) The protein expression of FoxO1, PGC-1α, HNF-4, PPAR-α, PEPCK, and G-6-Pase. (G) Knockdown of endogenous FOXO1 in the HepG2-IR cells decreased the glucose contents and co-cultivation of spexin did not further reduce glucose contents. * *P* < 0.05, # *P* > 0.05.

**Figure 6:**
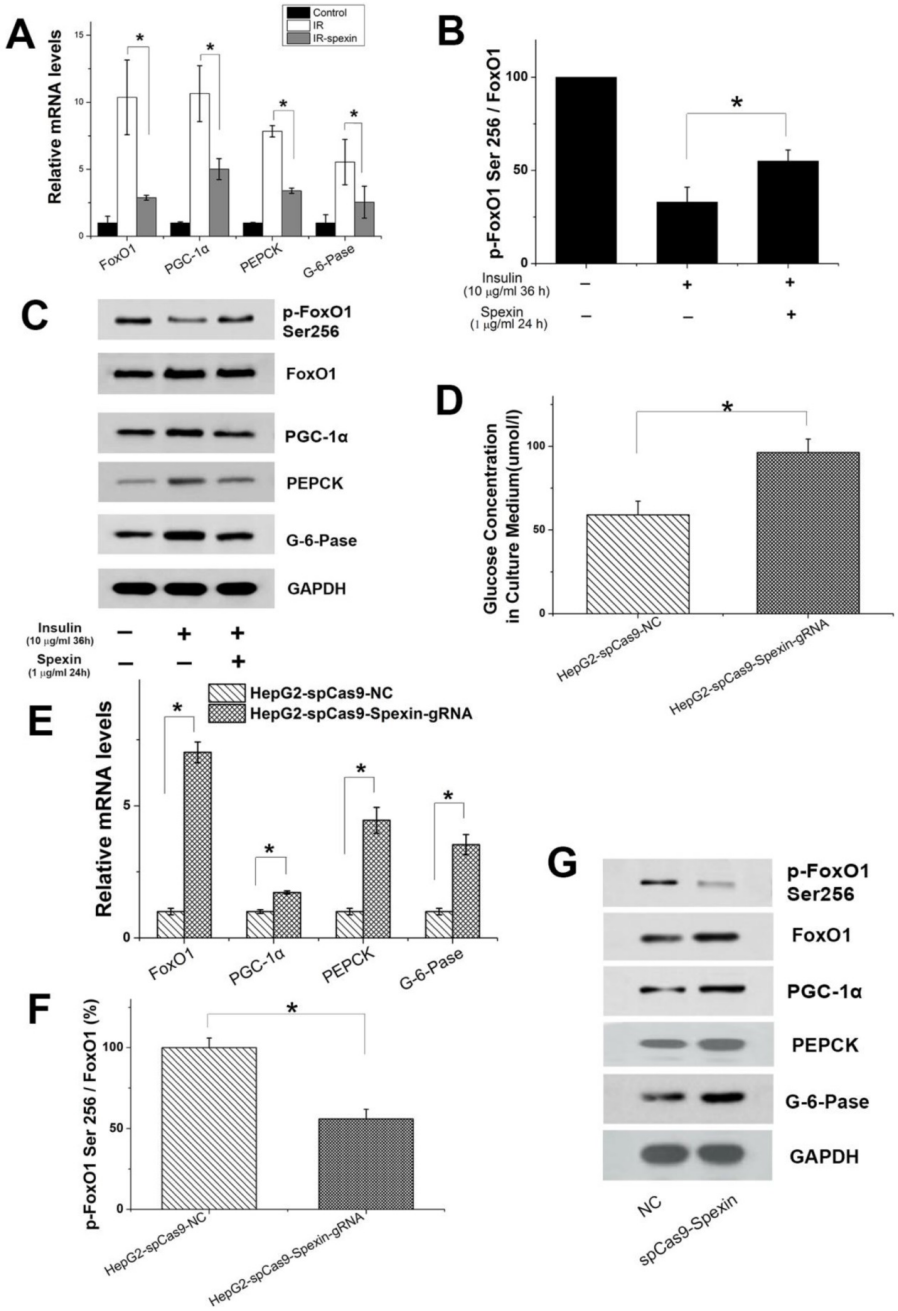
** Expression of important factors in gluconeogenesis in primary hepatocytes and spexin gene knockout activated the gluconeogenesis signalling pathway.** (A) Spexin inhibited the expression of FoxO1, PGC-1α, PEPCK, G-6-Pase mRNA in primary hepatocytes. (B) Spexin intervention increased the p-FoxO1 Ser256/FoxO1 ratio in primary hepatocytes. (C) Spexin inhibited the protein expression of FoxO1, PGC-1α, PEPCK, and G-6-Pase in primary hepatocytes. (D) The glucose concentration in the culture media of spexin gene knockout HepG2 cells was significantly increased. (E) The mRNA expression of FoxO1, PGC-1α, PEPCK, and G-6-Pase was significantly increased in spexin gene knockdown HepG2 cells. (F) Spexin gene knockdown decreased the Ser256/FoxO1 p-FoxO1 ratio in HepG2 cells. (G) The protein expression of FoxO1, PGC-1α, PEPCK, and G-6-Pase in spexin gene knockdown HepG2 cells was increased. * *P* < 0.05.

Spexin dose-dependently inhibited the mRNA expression of FoxO1, PGC-1α, PEPCK, and G-6-Pase (Fig. 5A, B, C, D). Treatment with 10 μg/mL spexin decreased FoxO1 mRNA expression by approximately 80%, PGC-1α mRNA expression by approximately 60%, PEPCK mRNA expression by approximately 85%, and G-6-Pase mRNA expression by approximately 75%. The protein expression of key transcription factors and key enzymes in the gluconeogenesis pathway were also measured by Western blotting. The spexin treatment reduced FoxO1, PGC-1α, PEPCK and G-6-Pase protein expression in a dose-dependent manner and increased the ratio of p-FoxO1 Ser256/FoxO1 (Fig. 5E, F). The protein expressions of HNF-4 and PPAR-α increased in HepG2-IR cells. But there was no significant change after spexin treatment (Fig. 5F). Knockdown of endogenous FOXO1 by siRNAs in HepG2-IR cells decreased the glucose contents in the supernatant, while co-cultivation of spexin did not further reduce glucose contents in the supernatant (Fig. 5G). We further validated the above conclusions in primary hepatocytes. Spexin incubation inhibited the mRNA and protein expression of FoxO1, PGC-1α, PEPCK and G-6-Pase and the p-FoxO1 Ser256/FoxO1 ratio was also increased in primary hepatocytes (Fig. 6A, B, C).

### CRISPR/Cas9-mediated disruption of spexin expression in HepG2 cells activated the gluconeogenesis signalling pathway

We hypothesized that gluconeogenesis should be inhibited when HepG2-IR cells are incubated with spexin if gluconeogenesis is the consequence of decreased spexin levels in HepG2-IR cells. Conversely, gluconeogenesis should increase after spexin expression is disrupted in HepG2 cells. HepG2 cells were cotransfected with a plasmid encoding spexin gene-specific guide RNAs and Cas9 and a plasmid encoding selection genes or transfected with the control CRISPR/Cas9 plasmid alone (control HepG2). The differences in expression were confirmed by ELISA and qRT-PCR (see Supplementary Fig S3A). CRISPR/Cas9-mediated disruption of spexin expression in HepG2 cells resulted in a significant increase of the glucose concentration in the medium (Fig. 6D). Notably, we found that suppression of spexin in HepG2 cells increased the expression of FoxO1, PGC-1α, PEPCK, and G-6-Pase mRNA and protein expression (Fig. 6E, G). In addition, CRISPR/Cas9-mediated disruption of spexin expression in HepG2 cells reduced the p-FoxO1 Ser256/FoxO1 ratio (Fig. 6F, G), indicating a decrease in FoxO1 phosphorylation and an increase of the interaction between FoxO1 and PGC-1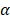 in the nucleus.

## Discussion

Previous studies showed that spexin may contribute to glucose metabolism 19, 27. The serum spexin levels were also significantly lower in obese children or patients with type 1 diabetes 28, 29. But a study of adolescents with T2DM did not report a difference in spexin as compared to controls 30. In our study, we found that the serum spexin levels were reduced in patients with T2DM (*P* < 0.01). Moreover, strong negative correlations were established between the serum spexin level and HOMA-IR values in the OGTT after adjusting for BMI. These results suggested that spexin might involve in insulin resistance. The effects of spexin on insulin resistance in a HFD-induced SD rat model were examined to further test the hypothesis. HFD feeding has been shown to affect glucose, impair insulin sensitivity, and induce metabolic dysfunction 31, 32. In our study, 8-week spexin treatment consistently resulted in weight loss, which is consistent with previous observations 20, 33. Weight loss can affect glucose control, serum FFA and other metabolic abnormalities. Due to the limited conditions, we failed to present whether the spexin injections could result in less food intake, impaired food absorption, or increased energy expenditure. We believe that spexin maybe has an effect on body weight while it also plays an important role in insulin resistance. Kolodziejski et al 34 report that spexin and kisspeptin show negative correlations with obesity, insulin resistance indices, and hormones known to affect insulin sensitivity in females. Ge et al 35 also find that spexin improves glucose tolerance, decreases insulin resistance and HbAlc in HFD-induced T2DM mice. We found that exogenous spexin also significantly reduced the insulin concentration and HOMA-IR value in HFD-fed rats. Therefore, the spexin was identified as a positive regulator of insulin sensitivity. We used HOMA-IR and hyperinsulinemic-euglycemic clamp studies to evaluate the effect of spexin on insulin resistance in HFD-induced rats. Exogenous spexin administration ameliorated HFD-induced metabolic dysfunction, as well as the serum insulin level and HOMA-IR value. As demonstrated by the hyperinsulinemic-euglycemic clamp test, the GIR was higher in the HFD + spexin group than in the HFD group at the clamp point (*P* < 0.05), indicating that exogenous spexin can improve insulin resistance in HFD-induced rats.

Insulin resistance primarily exerts its dysregulatory metabolic effects on the liver, which plays a central role in controlling carbohydrate, lipid, and protein metabolism. Indeed, the liver is a key metabolic organ that governs the body's energy metabolism. In the postprandial state, insulin reduces the blood glucose levels by stimulating glucose uptake in adipose tissues and muscles, as well as by inhibiting hepatic glucose production 36. Moreover, in the liver, insulin stimulates the conversion of excess glucose into glycogen (glycogenesis) and triacylglyceride (lipogenesis) for the long-term energy storage 37-39. In this study, the HGP of the HFD-induced rats was not completely inhibited (inhibition rate of only 26% at the steady-state hyperinsulinemic-euglycemic clamp), whereas the rate of HGP inhibition in the normal control group reached 80%. However, spexin intervention significantly decreased the rate of HGP inhibition to 58% in HFD-induced rats. These *in vivo* data showed that exogenous spexin inhibited hepatic glucose output in HFD-induced rats.

In the fasted state or during exercise, fuel substrates (e.g., glucose and fatty acids) are released from the liver into the circulation and are metabolized by muscle, adipose tissue, and other extrahepatic tissues. Adipose tissue produces and releases nonesterified fatty acids and glycerol via lipolysis. Muscle tissue breaks down the glycogen and proteins and releases lactate and alanine. Alanine, lactate, and glycerol are delivered to the liver and used as precursors to synthesize glucose (gluconeogenesis). Hepatic gluconeogenesis is the core phenomenon that provokes insulin resistance and is the main reason for hepatic glucose output 37. Using an insulin-resistant cell model, for the first time, we found that spexin dose- and time-dependently inhibited gluconeogenesis. Moreover, CRISPR/Cas9-mediated disruption of spexin expression in HepG2 cells caused a significant increase in gluconeogenesis.

Insulin resistance is a multifaceted disorder that involves the modulation of various genes at the transcriptional and translational levels. The rate of gluconeogenesis is determined by both the availability of gluconeogenic substrates and the expression/activation of gluconeogenic enzymes (e.g., PEPCK and G-6-Pase) that control key steps of gluconeogenesis. Numerous transcription factors, including FoxO1, CREB, and CCAAT/enhancer binding proteins α/β (C/EBPα/β), have been identified to stimulate the expression of PEPCK and G-6-Pase 40-43. CREB is critical in modulating the fasting-mediated transcriptional activation of PGC-1α, which serve as essential transcriptional regulators for the activation of gluconeogenic genes 44. FoxO1 is a well-documented gluconeogenic transcription factor that is co-activated with PGC-1α to stimulate the expression of PEPCK-C and G-6-Pase 45-48. FoxO1 deletion in the liver has been shown to curtail excess glucose production caused by generalized ablation of insulin receptors and to prevent neonatal diabetes and hepatosteatosis in insulin receptor knockout mice 49. Our data suggest that there is increased circulating insulin and insulin resistance under HFD feeding. In insulin resistance, Akt level decreased and CREB, nuclear FoxO1 expression is increased and co-activated with PGC-1α, in turn regulates downstream target genes to promote gluconeogenesis. Following spexin treatment in HFD-induced rats, the levels of phosphorylated FoxO1 in the cytoplasm were increased; co-activation with PGC-1α, and CREB was decreased. The proteins were ineffective at activating their target genes. These findings were consistent with the results of HepG2 model of insulin resistance. Future studies are warranted to explore the roles of upstream factors in the FoxO1 signal transduction pathway in insulin resistance.

Insulin resistance is a determinant for the development of obesity or T2DM and also contributes to the pathogenesis of non-alcoholic fatty liver disease (NAFLD). NAFLD is also associated with increased gluconeogenesis in humans 50. Free fatty acids (FFA) are thought to inhibit glucose use in part by disrupting components of the insulin signaling cascade in liver. Using an animal model, it was shown that spexin reduced serum FFA levels and attenuated the hepatocyte steatosis in HFD-fed mice. However, the molecular mechanisms underlying remain elusive.

In summary, to the best of our knowledge, the results of the present study shows, for the first time, that spexin improves insulin resistance in HFD-induced rats and insulin resistant cells. This regulation may involve the control of gluconeogenesis and a reduction of hepatic steatosis through the FoxO1/PGC-1α pathway. Thus, our data indicate a potential therapeutic benefit of spexin in regulating insulin resistance in metabolic diseases, although additional long-term studies are needed to confirm this beneficial effect.

## Supplementary Material

Supplementary figures and tables.

## Figures and Tables

**Table 1:** Parameters of glucose metabolism and correlations with the serum spexin level in the T2DM group and control group.

	T2DM	Control	*P* value	Correlation with the serum spexin level	
				*r*	*P* value
Numbers	23	22			
Age (years)	55.3 ± 13.2	52.7 ± 17.4	0.725	-0.011	0.941
BMI (kg/m^2^)	23.5 ± 2.8	22.3 ± 2.6	0.169	-0.254	0.092
FBG (mmol/L)	9.2 ± 1.5	4.6 ± 0.5	<0.001	-0.583	<0.001
PBG1h (mmol/L)	14.6 ± 3.4	7.2 ± 1.1	<0.001	-0.457	0.002
PBG2h (mmol/L)	15.3 ± 4.1	6.6 ± 1.1	<0.001	-0.502	0.001
HbA1c (%)	9.5 ± 2.1	5.6 ± 0.4	<0.001	-0.431	0.004
FINS (μIU/mL)	11.9 ± 3.4	10.3 ± 2.8	0.080	-0.132	0.400
PINS1h (μIU/mL)	64.5 ± 22.6	63.8 ± 29.6	0.488	0.155	0.321
PINS2h (μIU/mL)	71.9 ± 25.3	45.9 ± 22.6	0.001	-0.180	0.248
TG (mmol/L)	2.01±0.71	1.28±0.61	0.001	-0.191	0.209
TC (mmol/L)	5.15±0.98	4.66±0.86	0.085	-0.180	0.237
LDL-C (mmol/L)	3.57±0.96	2.73±0.78	0.002	-0.270	0.072
HDL-C (mmol/L)	1.02±0.24	1.22±0.62	0.156	0.033	0.829
Serum spexin (ng/mL)	1.69 ± 1.07	3.82 ± 1.54	<0.001		
HOMA-IR	4.85 ± 1.68	2.12 ± 0.58	<0.001	-0.498	0.001

The data are presented as the means ± SD for continuous data. FINS, PINS1h and PINS2h were log-transformed before performing the correlation analysis.

## References

[1] Milburn MV, Lawton KA (2013). Application of metabolomics to diagnosis of insulin resistance. Annu Rev Med.

[2] Weyer C, Bogardus C, Mott DM (1999). The natural history of insulin secretory dysfunction and insulin resistance in the pathogenesis of type 2 diabetes mellitus. J Clin Invest.

[3] Monnier L, Colette C, Dunseath GJ (2007). The loss of postprandial glycemic control precedes stepwise deterioration of fasting with worsening diabetes. Diabetes Care.

[4] Rizza RA (2010). Pathogenesis of fasting and postprandial hyperglycemia in type 2 diabetes: implications for therapy. Diabetes.

[5] Lin HV, Accili D (2011). Hormonal regulation of hepatic glucose production in health and disease. Cell Metab.

[6] Barthel A, Schmoll D (2003). Novel concepts in insulin regulation of hepatic gluconeogenesis. Am J Physiol Endocrinol Metab.

[7] Nordlie RC, Foster JD, Lange AJ (1999). Regulation of glucose production by the liver. Annu Rev Nutr.

[8] Tsintzas K, Norton L, Chokkalingam K (2013). Independent and combined effects of acute physiological hyperglycaemia and hyperinsulinaemia on metabolic gene expression in human skeletal muscle. Clin Sci (Lond).

[9] Sugden MC, Caton PW, Holness MJ (2010). PPAR control: it's SIRTainly as easy as PGC. J Endocrino.

[10] Mouchiroud L, Eichner LJ, Shaw RJ (2014). Transcriptional coregulators: fine-tuning metabolism. Cell Metab.

[11] Yoon JC, Puigserver P, Chen G (2001). Control of hepatic gluconeogenesis through the transcriptional coactivator PGC-1. Nature.

[12] Accili D, Arden KC (2004). FoxOs at the crossroads of cellular metabolism, differentiation, and transformation. Cell.

[13] Kops GJ, de Ruiter ND, De Vries-Smits AM (1999). Direct control of the Forkhead transcription factor AFX by protein kinase B. Nature.

[14] Lu M, Wan M, Leavens KF (2012). Insulin regulates liver metabolism in vivo in the absence of hepatic Akt and Foxo1. Nat Med.

[15] Marinho R, Mekary RA, Munoz VR (2015). Regulation of hepatic TRB3/Akt interaction induced by physical exercise and its effect on the hepatic glucose production in an insulin resistance state. Diabetol Metab Syndr.

[16] Mirabeau O, Perlas E, Severini C (2007). Identification of novel peptide hormones in the human proteome by hidden Markov model screening. Genome Res.

[17] Wan B, Wang XR, Zhou YB (2010). C12ORF39, a novel secreted protein with a typical amidation processing signal. Biosci Rep.

[18] Porzionato A, Rucinski M, Macchi V (2010). Spexin expression in normal rat tissues. J Histochem Cytochem.

[19] Gu L, Ma Y, Gu M (2015). Spexin peptide is expressed in human endocrine and epithelial tissues and reduced after glucose load in type 2 diabetes. Peptides.

[20] Walewski JL, Ge F, Lobdell Ht (2014). Spexin is a novel human peptide that reduces adipocyte uptake of long chain fatty acids and causes weight loss in rodents with diet-induced obesity. Obesity (Silver Spring).

[21] Albareda M, Rodriguez-Espinosa J, Murugo M (2000). Assessment of insulin sensitivity and beta-cell function from measurements in the fasting state and during an oral glucose tolerance test. Diabetologia.

[22] Onishi Y, Honda M, Ogihara T (2003). Ethanol feeding induces insulin resistance with enhanced PI 3-kinase activation. Biochem Biophys Res Commun.

[23] James DE, Burleigh KM, Kraegen EW (1986). In vivo glucose metabolism in individual tissues of the rat. Interaction between epinephrine and insulin. J Biol Chem.

[24] Marc N, Galisteo M, Lagadic-Gossmann D (2000). Regulation of phenobarbital induction of the cytochrome P450 2b9/10 genes in primary mouse hepatocyte culture. Involvement of calcium- and cAMP-dependent pathways. Eur J Biochem.

[25] Xie W, Wang W, Su H (2006). Effect of ethanolic extracts of Ananas comosus L. leaves on insulin sensitivity in rats and HepG2. Comp Biochem Physiol C Toxicol Pharmacol.

[26] Zhang HJ, Ji BP, Chen G (2009). A combination of grape seed-derived procyanidins and gypenosides alleviates insulin resistance in mice and HepG2 cells. J Food Sci.

[27] Al-Daghri NM, Alenad A, Al-Hazmi H (2018). Spexin Levels Are Associated with Metabolic Syndrome Components. Dis Markers.

[28] Karaca A, Bakar-Ates F, Ersoz Gulcelik N (2018). Decreased Spexin Levels in Patients with Type 1 and Type 2 Diabetes. Med Princ Pract.

[29] Kumar S, Hossain J, Nader N (2016). Decreased Circulating Levels of Spexin in Obese Children. J Clin Endocrinol Metab.

[30] Hodges SK, Teague AM, Dasari PS (2018). Effect of obesity and type 2 diabetes, and glucose ingestion on circulating spexin concentration in adolescents. Pediatr Diabetes.

[31] Qatanani M, Lazar MA (2007). Mechanisms of obesity-associated insulin resistance: many choices on the menu. Genes Dev.

[32] de Luca C, Olefsky JM (2008). Inflammation and insulin resistance. FEBS Lett.

[33] Wong MK, Sze KH, Chen T (2013). Goldfish spexin: solution structure and novel function as a satiety factor in feeding control. Am J Physiol Endocrinol Metab.

[34] Kolodziejski PA, Pruszynska-Oszmalek E, Korek E (2018). Serum levels of spexin and kisspeptin negatively correlate with obesity and insulin resistance in women. Physiol Res.

[35] Ge JF, Walewski JL, Anglade D (2016). Regulation of Hepatocellular Fatty Acid Uptake in Mouse Models of Fatty Liver Disease with and without Functional Leptin Signaling: Roles of NfKB and SREBP-1C and the Effects of Spexin. Semin Liver Dis.

[36] Puigserver P, Rhee J, Donovan J (2003). Insulin-regulated hepatic gluconeogenesis through FOXO1-PGC-1alpha interaction. Nature.

[37] Moller DE (2001). New drug targets for type 2 diabetes and the metabolic syndrome. Nature.

[38] Wong RH, Chang I, Hudak CS (2009). A role of DNA-PK for the metabolic gene regulation in response to insulin. Cell.

[39] Wang Y, Wong RH, Tang T (2013). Phosphorylation and recruitment of BAF60c in chromatin remodeling for lipogenesis in response to insulin. Mol Cell.

[40] Tikhanovich I, Cox J, Weinman SA (2013). Forkhead box class O transcription factors in liver function and disease. J Gastroenterol Hepatol.

[41] Xiong X, Tao R, DePinho RA (2013). Deletion of hepatic FoxO1/3/4 genes in mice significantly impacts on glucose metabolism through downregulation of gluconeogenesis and upregulation of glycolysis. PLoS One.

[42] Oh KJ, Han HS, Kim MJ (2013). CREB and FoxO1: two transcription factors for the regulation of hepatic gluconeogenesis. BMB Rep.

[43] Qiao L, MacLean PS, You H (2006). knocking down liver ccaat/enhancer-binding protein alpha by adenovirus-transduced silent interfering ribonucleic acid improves hepatic gluconeogenesis and lipid homeostasis in db/db mice. Endocrinology.

[44] Herzig S, Long F, Jhala US (2001). CREB regulates hepatic gluconeogenesis through the coactivator PGC-1. Nature.

[45] Haeusler RA, Hartil K, Vaitheesvaran B (2014). Integrated control of hepatic lipogenesis versus glucose production requires FoxO transcription factors. Nat Commun.

[46] Shin DJ, Joshi P, Hong SH (2012). Genome-wide analysis of FoxO1 binding in hepatic chromatin: potential involvement of FoxO1 in linking retinoid signaling to hepatic gluconeogenesis. Nucleic Acids Res.

[47] Liang H, Balas B, Tantiwong P (2009). Whole body overexpression of PGC-1alpha has opposite effects on hepatic and muscle insulin sensitivity. Am J Physiol Endocrinol Metab.

[48] Bernsmeier C, Calabrese D, Heim MH (2014). Hepatitis C virus dysregulates glucose homeostasis by a dual mechanism involving induction of PGC1alpha and dephosphorylation of FoxO1. J Viral Hepat.

[49] Matsumoto M, Pocai A, Rossetti L (2007). Impaired regulation of hepatic glucose production in mice lacking the forkhead transcription factor Foxo1 in liver. Cell Metab.

[50] Sunny NE, Parks EJ, Browning JD (2011). Excessive hepatic mitochondrial TCA cycle and gluconeogenesis in humans with nonalcoholic fatty liver disease. Cell Metab.

