# Supplementary material for: Spexin alleviates insulin resistance and inhibits hepatic gluconeogenesis via the FoxO1/PGC-1α pathway in high-fat-diet-induced rats and insulin resistant cells
Source: Int J Biol Sci. 2019 Nov 1;15(13):2815–29. doi: 10.7150/ijbs.31781 (PMC6909969; doi:10.7150/ijbs.31781)
Supplement: Supplementary file 1 — Supplementary figures and tables. [file ijbsv15p2815s1.pdf]

Spexin alleviates insulin resistance and inhibits hepatic gluconeogenesis via the FoxO1/PGC-1α pathway in high-fat-diet-induced rats and insulin-resistant cells

Liping Gu[1*], Xiaoying Ding[1*], Yufan Wang[1], Mingyu Gu[1], Jielei Zhang[1], Shuai Yan[1], Na Li[1], Zhiyi Song[1], Jiajing Yin[1], Leilei Lu[23], and Yongde Peng[1]

[1] Department of Endocrinology and Metabolism, Shanghai General Hospital, Shanghai Jiao Tong University School of Medicine, Shanghai, China. [2] Shanghai Intertek Medical diagnostic Testing Center Co; Ltd, Shanghai 200436, China. [3] School of Pharmaceutical Engineering& Life Science, Changzhou University, Changzhou, 213164 China. * These authors contributed equally to this study. Correspondence and requests for materials should be addressed to Yong-de Peng (E-mail: pengyongde0908@126.com)

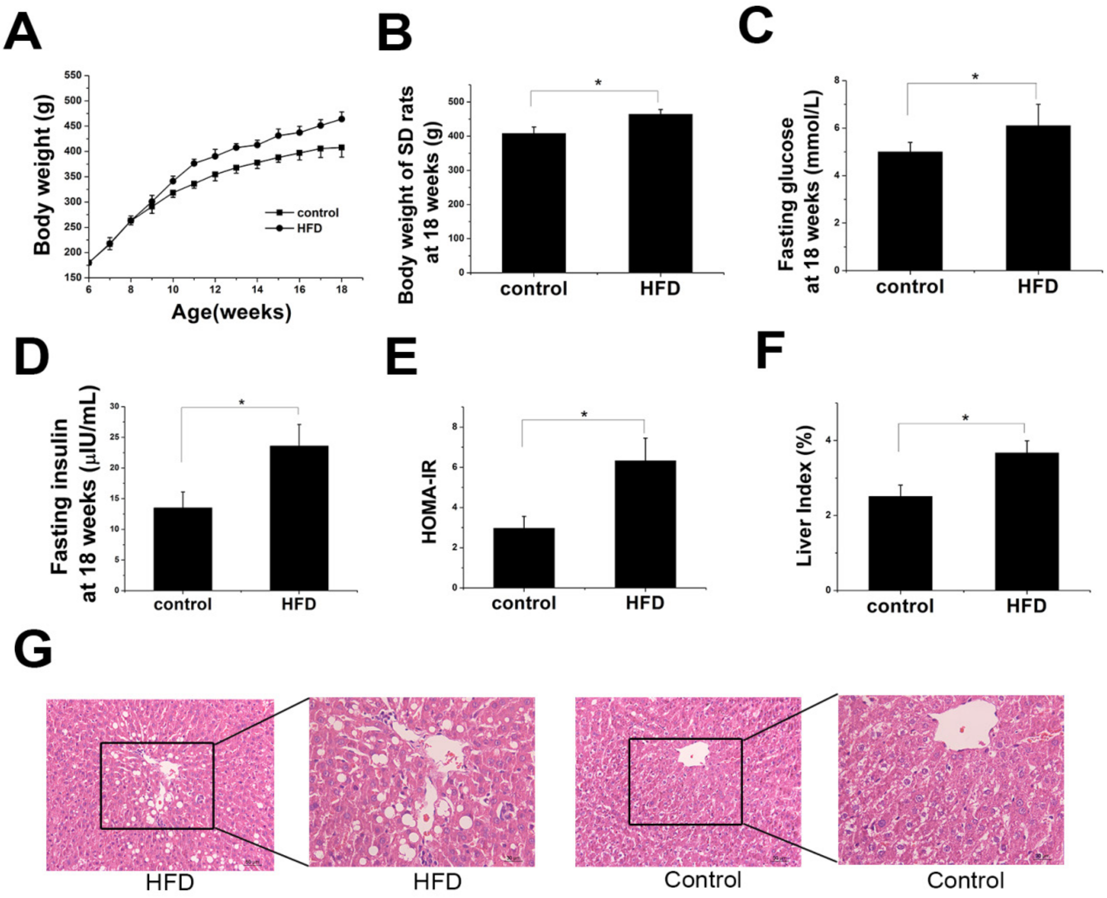

Supplementary Figure 1. Comparison of body weight, blood parameters, and HOMA-IR in HFD-induced SD rats and control rats at 18 weeks old. (A) The changes of body weight from 0 to 18 weeks in HFD-induced rats and control group. (B) Body weight in HFD group was significantly higher than control group at 18 weeks (* $P < 0.05$). (C) Fasting plasma glucose in HFD group was significantly higher than control group at 18 weeks (* $P < 0.05$). (D) Fasting insulin level in HFD group was significantly higher than control group at 18 weeks (* $P < 0.05$). (E) HOMA-IR in HFD group was significantly higher than control group at 18 weeks (* $P < 0.05$). (F) The liver index in HFD group was significantly higher than that in control group (* $P < 0.05$). (G) Liver morphology observed by H&E staining in HFD group and control group. Liver cells in the HFD group showed cell enlargement, diffused hepatic steatosis, and obvious fat infiltration. In control group, the liver cells showed a compact structure, clear edge, and normal size, with a large and round cell nucleus, even cytoplasm, and no lipid droplets.

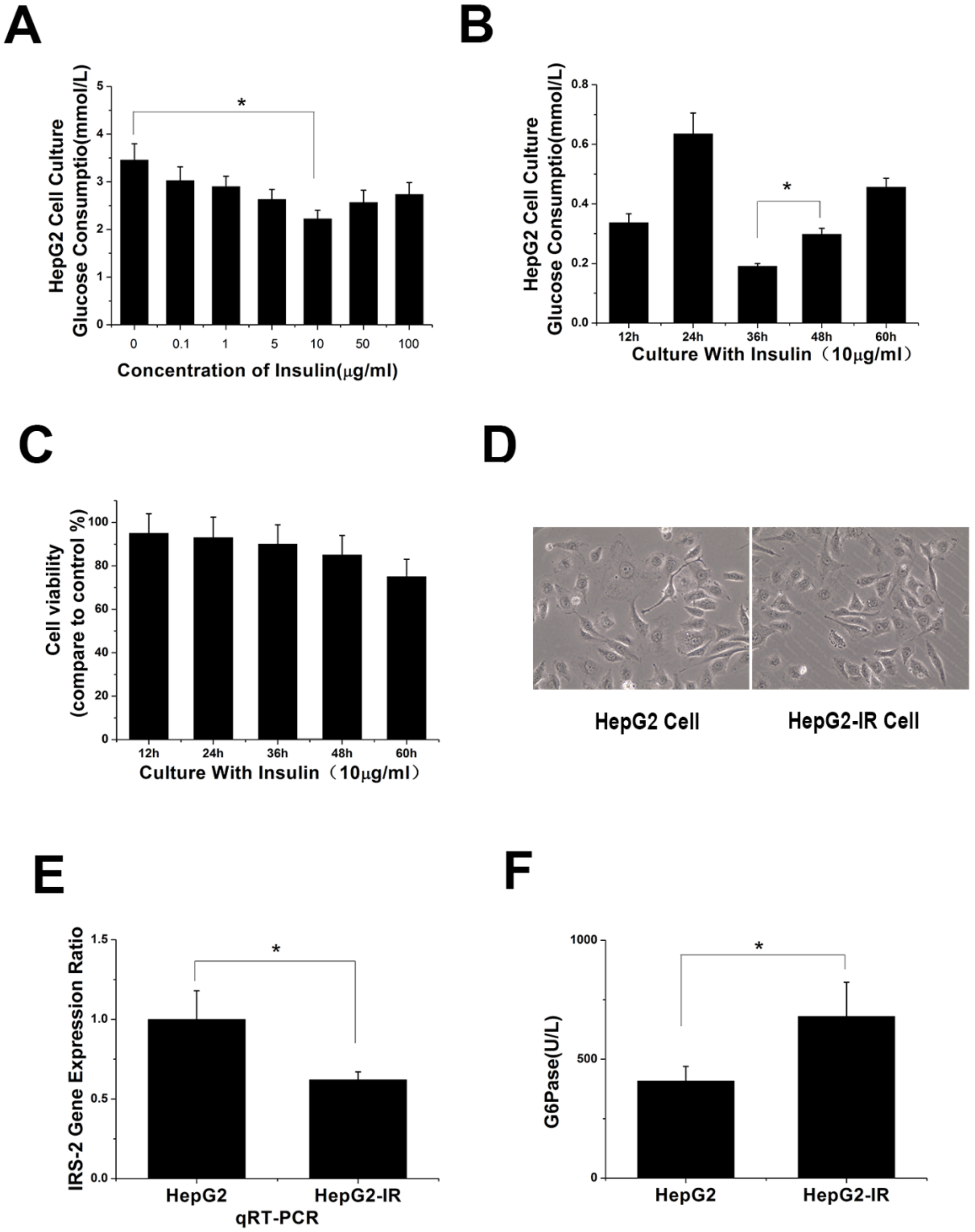

Supplementary Figure 2. HepG2 cells cultured with 10 μg/ml insulin for 36 h could establish a stable and successful insulin resistant model of HepG2 cells. (A) Different concentrations of insulin treated HepG2 cells for 36 h, and glucose consumption in the culture of 10 μg/ml insulin group was the smallest. (B) HepG2 cells were cultured with 10 μg/ml insulin for 12 h, 24 h, 36 h, 48 h, and 60 h. Glucose consumption in the supernatant in 36 h group was the smallest. (C) No significant difference in cell viability was detected by CCK-8 assay after 36 h treatment of high

concentration of insulin. (D) No difference in morphology of HepG2 cells and HepG2-IR cells was observed under the optical microscope. (E) Expression of IRS-2 mRNA detected by qRT-PCR showed a significant decrease in HepG2-IR cells (* $P < 0.05$). (F) Expression of G-6-Pase in HepG2-IR cell culture was significantly increased detected by ELISA (* $P < 0.05$).

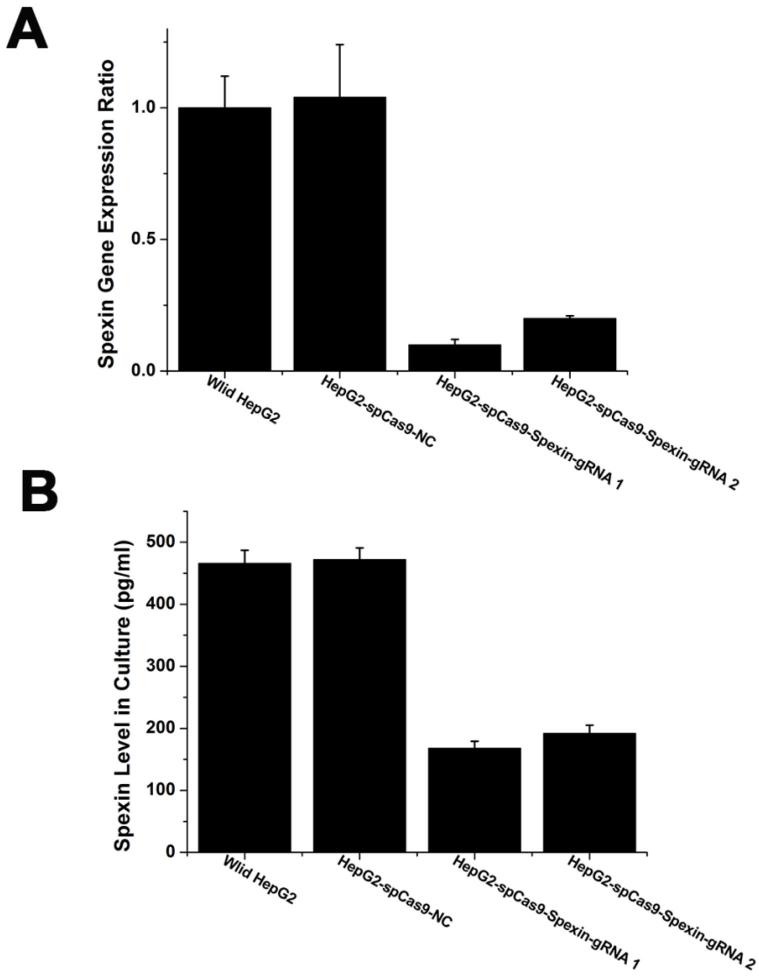

Supplementary Figure 3. CRISPR/Cas9-mediated disruption of spexin expression in HepG2 cells. (A) Compared with the blank group and the HepG2-spCas9-NC group, the cell expression of spexin mRNA in HepG2-spCas9-Spexin-gRNA group was significantly lower (* $P < 0.05$). (B) The protein expression of spexin in cell culture was detected by ELISA. The level of spexin expression in HepG2-spCas9-Spexin-gRNA culture was significantly lower than that in blank group and HepG2-spCas9-NC group (* $P < 0.05$).

Supplementary Table 1A. Primer sequences for qRT-PCR.

Gene Name Primer	Species	Primer Sequence（5-3）
PGC-1α	Human	F:AGGCAAGCAAGCAGGTCT<br>R:GTCATCAAACAGGCCATCC
	Rat	F: GGAGCTGGATGGCTTGGGACAT<br>R: TTCGCAGGCTCATTGTTGTAC
FoxO1	Human	F: TGGACATGCTCAGCAGACATC<br>R: TTGGGTCAGGCGGTTCA
	Rat	F: CAGCAAATCAAGTTATGGAGGA<br>R: TATCATTGTGGGGAGGAGAGTC
PEPCK	Human	F: GGTTCCCTGGGTGCATGAAA<br>R: CACGTAGGGTGAATCCGTCAG
	Rat	F: GAGATCATCTCCTTCGGAAGCG<br>R: TTAGTTATGCCCAGGATCAGCATG
G-6-Pase	Human	F: GACCTCAGGAATGCCTTCTACG<br>R: AGTCAGTATCCAAAACCCACCAG
	Rat	F: AACGTCTGTCTGTCCCGGATCTAC<br>R: ACCTCTGGAGGCTGGCATTG
GAPDH	Human	F: ATGGGGAAGGTGAAGGTCG<br>R: GGGGTCATTGATGGCAACAATA
β-actin	Rat	F: ACCCACACTGTGCCCATCTATG<br>R: AATGTCACGCACGATTTCCCT
